# Decades-old studies of fungi associated with mammalian lungs and modern DNA sequencing approaches help define the nature of the lung mycobiome

**DOI:** 10.1371/journal.ppat.1008684

**Published:** 2020-07-30

**Authors:** Paris S. Hamm, John W. Taylor, Joseph A. Cook, Donald O. Natvig

**Affiliations:** 1 Department of Biology, University of New Mexico, Albuquerque, New Mexico, United States of America; 2 Department of Plant and Microbial Biology, University of California, Berkeley, California, United States of America; 3 Museum of Southwestern Biology, University of New Mexico, Albuquerque, New Mexico, United States of America; McGill University, CANADA

## Introduction

The vertebrate lung is the organ with the largest surface area presented to the external environment. The combined alveolar surface area of both adult human lungs is about 100 m^2^ [1], and typically 10,000 to 20,000 liters of air are inhaled per day [2]. Coupled with the fact that fungal spore densities of 10 to 50 spores per liter of air are common [3, 4], the average person inhales up to 100,000 or more fungal spores daily. Lung surface areas and inhalation volumes for small mammals are comparable to those of humans when scaled for size. Moreover, many small mammals live in microenvironments (notably burrows and understories) where they are exposed to high densities of airborne spores derived from the growth of fungi on substrates in soil and litter. While lung tissues have physical and immunological defenses against infection, it is also the case that the air and blood-vessel interface is by necessity fragile. Taken together, these factors make it unsurprising that many fungi have adaptations that permit commensal, pathogenic, and perhaps, yet to be discovered, mutualistic relationships with lungs.

The title of this article references the fact that the study of interactions between lungs and certain fungi began in the first half of the 20th century. As we discuss here, modern molecular methods combined with culture-based approaches are revealing that lung mycobiomics is a field rich in opportunities for studying interactions of fungi across the vertebrate tree of life. Moreover, next-generation sequencing efforts now provide important new contexts for the study of lung-inhabiting fungi that began more than seven decades ago. Here, we briefly cover the historical context of the lung mycobiome, discuss future directions and questions, and propose that the lung mycobiome may form a reservoir for opportunistic fungal mycoses caused by commensal lung-adapted fungi. We discuss the following points: (1) Human lungs have a mycobiome. (2) Lungs of nonhuman mammals commonly possess a diversity of fungi. (3) Studies of fungi associated with lungs have implications for the ecology, distribution, and pathogenicity of specific fungi, especially members of the Onygenales and species of *Pneumocystis*. (4) A framework is needed to distinguish among lung-adapted fungi (some of which can be opportunistic pathogens), members of the general mammalian mycobiome, and fungi that are present in lungs because of incidental inhalation. (5) Museum research collections provide an important resource for addressing these issues.

## Human lungs possess a mycobiome

The Human Microbiome Project launched by the National Institutes of Health (NIH) in 2007 ultimately resulted in recognition that microbes can alter the physiology, immunity, and neurological development of their hosts [5]. The traditional thought that lung tissues are sterile, left unchallenged by the difficulties of sampling lungs, resulted in lung microbiome studies lagging other aspects of human microbiomics. Innovations in specimen collection and advances in deep sequencing and bioinformatics have now established a genuine lung microbiome [6]. Studies of the lung microbiome have focused primarily on bacteria, but studies of the fungal component of the lung microbiome (mycobiome) are beginning to emerge.

Initial investigations of the human lung mycobiome involved individuals with lung diseases such as cystic fibrosis (CF) [7], asthma, and chronic obstructive pulmonary disease. From these studies, it appears that the mycobiome of unhealthy lungs can become dominated by one or few fungal species but may have a greater fungal burden [8]. Species of *Candida*, especially *Candida albicans*, can be dominant fungi in the lungs of CF patients and are associated with reduced lung function [9, 10]. Species of *Malassezia* were broadly detected in CF samples but at levels 10-fold to 50-fold lower than *Candida* [11]. *Malassezia* species have similarly been found in the lungs of asthmatic patients [12] and healthy individuals [8] and may be commonplace in the lungs. In addition, dominant fungal taxa associated with the lungs of healthy individuals include species of *Cladosporium*, *Aspergillus*, and *Penicillium* [12, 13]. Analyses of sputum samples from both diseased and healthy human airways have indicated a fungal community composed largely of transient species, suggesting that the majority of fungi in such samples represent recent inhalation rather than colonization [14, 15]. It is possible, however, that fungal colonization of airways is somewhat stochastic, resulting in the appearance of transient inhalation effects when colonization has in fact occurred. It is also the case that sputum samples might fail to sample fungi that are deeply invasive in lung tissue.

Several fungi deserve special attention in the context of lungs. These include species of *Pneumocystis*, which are obligate lung commensals detected in 20 to 60% of human lungs [16, 17], *Aspergillus fumigatus*, which is the primary cause of aspergillosis, and members of the order Onygenales. Medically important members of the Onygenales include species of *Coccidioides*, *Paracoccidioides*, *Blastomyces*, *Emmonsia*, and *Histoplasma* as well as members of a newly described genus, *Emergomyces* [18, 19]. While members of these groups are often associated with some type of pathology, it is now a legitimate question whether many of them are part of a normal lung mycobiome.

## Into the wild: Fungi in the lungs of nonhuman mammals

While the full mycobiome of nonhuman mammals has not been studied, such studies hold substantial promise for investigating the diversity and adaptations of fungi that occur in mammalian lungs. More than one-half century before interest in the human microbiome, several scientists systematically explored the microbiology of the vertebrate lung in rodents, rabbits, and carnivores in the context of pulmonary diseases. These studies strongly influenced our understanding of lung pathogens, their distributions, and life cycles. What these early studies missed was the fact that the lung is a microenvironment rich in microorganisms, many of which appear to be adapted to persist there. Similarly, mycobiome studies of humans stand to be informed by studies of wild rodents and other nonhuman mammals, in part because of limitations of sampling and sequencing approaches available for human studies (reviewed by [20]), but also because comparative analyses can help reveal long-term coevolutionary relationships.

**Fig 1:**
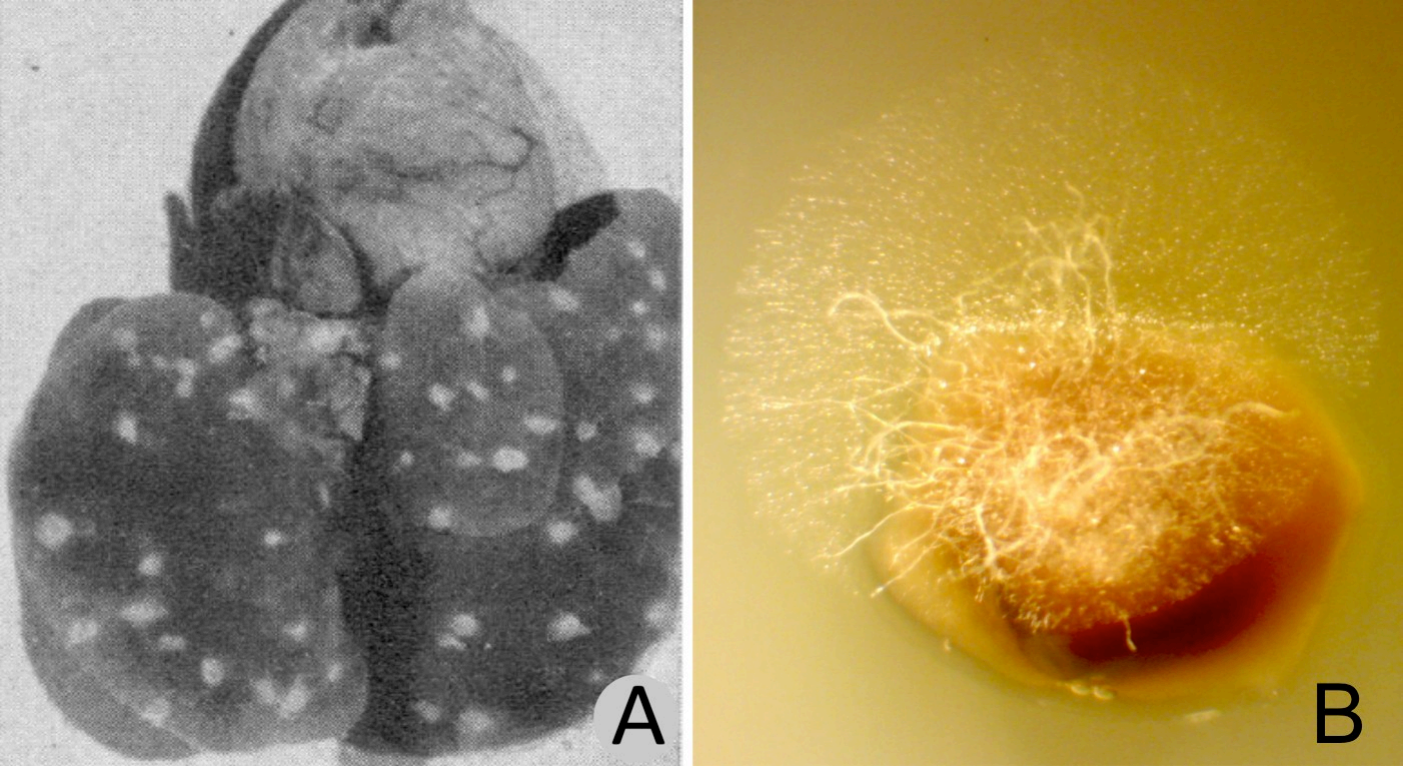
Small-mammal lung tissues showing fungal growth. (A) Lung from *Peromyscus* sp. collected in the vicinity of San Carlos, Arizona, with fungal lesions believed to be *Emmonsia parva* (*Blastomyces parvus*), reproduced from Emmons and Ashburn [21]. (B) Fungal hyphae in lung tissue from apparently healthy *Dipodomys heermanni* collected in Kern County, California, (MVZ:239394) from which *E*. *parva* was recovered in pure culture. The lung fragment shown was incubated for 48 hours on water agar with tetracycline (10 mg/ml) and chloramphenicol (50 mg/ml). The fungal growth shown in B is typical for small-mammal lungs we have examined from diverse species. Segments of any given lung plated on growth medium will often result in growth of multiple fungal species. The image shown in A is from *Public Health Reports* (volume 57) and is in the public domain.

In the 1940s and 1950s, culture-based and histopathological studies led by Chester Emmons identified and characterized two fungi from the lungs of mammals in Arizona (Fig 1). These fungi were known at the time as *Haplosporangium parvum* and *Coccidiodies immitis* [21]. These studies were followed by additional studies of *H*. *parvum* across the United States and Canada [22, 23], resulting in the recovery of pulmonary fungi from a number of deer mice (*Peromyscus*), pocket mice (*Perognathus* and *Chaetodipus*), woodrats (*Neotoma*), red squirrel (*Tamiasciurus*), beaver (*Castor canadensis*), cottontail rabbit (*Sylvilagus*), pika (*Ochotona princeps*), skunk (*Mephitis*), marten (*Martes americana*), and weasels (*Mustela frenata* and one *M*. *erminea*) [21–23]. *Haplosporangium parva* was later renamed *Emmonsia parva* [24] and, recently, is considered as a member of the genus *Blastomyces* (as *Blastomyces parvus*) [18]. *C*. *immitis* was later divided into two species, *C*. *immitis* and *C*. *posadasii*, with *C*. *posadasii* most common in Arizona and other locations outside California [25].

Onygenealan fungi emerged as presumed highly adapted pathogens of animal hosts approximately 150 million years ago [26]. Most or all appear capable of saprobic growth at some stage of their life cycle, ultimately producing spores that can be inhaled by a susceptible host, then switching to pathogenic forms (yeast-like stages, spherules, and adiaspores). Recently, Taylor and Barker [27] reviewed modern studies that support Emmons’ hypothesis [28, 29] that small mammals provide an environmental reservoir for species of *Coccidioides* as an endozoan commensal that then becomes a saprobe taking advantage of soil disturbance for wind dispersal. This hypothesis is supported in part by the fact that *Coccidioides* genomes have a reduced number of genes associated with plant cell-wall degradation and an increased number of genes associated with animal pathogenesis [26]. The hypothesis is also supported by growth studies of a close *Coccidioides* relative, *Uncinocarpus reesei*, which both shares the gene family expansions and contractions seen in *Coccidioides* and exhibits a preference for proteinaceous growth substrates over carbohydrates [30].

Historical and recent studies suggest that this hypothesis should be expanded to include other members of the Onygenales, such as *E*. *parva* (*B*. *parvus*) and members of the genus *Emergomyces* as well as other fungi that appear to be adapted for the lung mycobiome, including the special case of species of *Pneumocystis*. Because many of these fungi are known for their ability to cause opportunistic infections, from one point of view they could be considered to be commensal organisms with the potential to become pathogenic in hosts that become immune compromised or are otherwise weakened by comorbidities. An alternative interesting possibility is that these fungi are actually part of a community of organisms that coexist with host tissues and provide defenses against other infectious agents. In this context we note that although members of the Onygenales appear not to have been extensively studied in terms of antimicrobial compounds, reports of biologically active secondary metabolites, including compounds with antifungal activities, do exist [e.g., 31].

Perhaps the most exquisitely adapted members of lung mycobiomes, species of *Pneumocystis* are widespread among mammals, are specifically adapted to lung tissues, and exhibit host specificity. Co-evolution between mammalian hosts and species of *Pneumocystis* has been shown for humans, nonhuman primates, and bats [32–34]. Studies of such *Pneumocystis*-host associations have the potential to provide insights for transmission, phylogenetic relationships, and cell biology.

## A proposed framework for thinking about the lung mycobiome

Among the fungi observed in lung tissues, it is possible to conceive of three broad categories of fungi found in lungs of healthy mammals: (1) fungal cells that result from incidental inhalation, arguably not part of the true mycobiome; (2) fungi adapted to be part of the normal mammalian mycobiome but are not specialized for specific tissues; and 3) fungi evolved to inhabit lung tissues, whether as commensals, mutualists, or pathogens. Fungi that produce abundant quantities of airborne spores, such as the conidia of *Cladosporium*, *Aspergillus*, and *Penicillium*, or basidiospores of Agaricales [35, 36], are candidates for the first group. The second group might well include species of *Candida* and *Malassezia*, which are common members of the human mycobiome occurring in association with skin [37–39]. Species of *Pneumocystis*, long evolved symbionts that can be viewed as either commensals or opportunistic pathogens, easily belong in the third category; and multiple members of the Onygenales likely belong there as well. When the immune system of the human host is suppressed or compromised (e.g., asthmatics, CF, cancer treatment, and organ transplant), fungi that normally inhabit group one, for example *A*. *fumigatus*, can become life-threatening pathogens and constitute an additional group of opportunistic lung pathogens [40]. Teasing apart the differences and overlaps among these categories in wild animals can at least begin with molecular surveys that determine which fungal sequences are recovered repeatedly from lungs of animals. Ultimately, it will be valuable to compare the results from such surveys with those obtaining airborne fungi in the same geographic area, and with those of different host characteristics (species, age, sex, diet, genetics, and comorbidities) to understand fungal–host community interactions.

## Museum collections will prove important

Natural history collections are proving extremely useful to the goal of characterizing the mammalian lung mycobiome. More generally, these collections represent essential infrastructure for research, training, and education that continue to play vital roles in long-established fields (biodiversity discovery, systematics, and evolution), while now contributing to new research areas (genomics, stable isotopes, and pathogen research [41]). Next-generation sequencing paired with culture-based approaches and modern methods in cell biology allow a holistic approach for studying the mycocosm of the lung and for discovery of a diverse array of parasites and zoonotic pathogens. Museum collections can help address mycobiome questions related to spatial or temporal changes, community composition, host specificity (e.g., species), and individual variation (e.g., age, sex, diet, genetics, and comorbidities). For example, the geographical and chronological emergence of the chytrid fungus *Batrachochytrium dendrobatidis* in amphibians was documented using museum collections [42]. Likewise, museum specimens of bats collected prior to the emergence of white-nose syndrome caused by the fungus *Pseudogymnoascus destructans* have provided insights into the history [43]. The high frequency of detection of *Pneumocystis* in the autopsied lungs of humans [17] supports the hypothesis that frozen lung samples from nonhuman specimens in museum collections will prove valuable to studies of *Pneumocystis* and other members of the lung mycobiome. In this context we note that the Museum of Southwestern Biology at the University of New Mexico and the Museum of Vertebrate Zoology at the University of California, Berkeley, hold large ultrafrozen tissue collections of wild mammals [44] that are facilitating new avenues of research in pathobiology [45–47].

## Can’t we all get a lung: Conclusions and questions for the future

Recent studies point to the existence of a lung microbiome with a mycobiome component. The lung mycobiome community in small mammals appears to include, typically or often, members of the Onygenales, a conclusion where modern sequencing studies and decades-old culture studies now meet. Many questions remain: How do lung mycobiomes differ across mammalian species and larger vertebrate groups (mammals versus birds versus amniotes)? What roles do geography and climate play? Where are fungi located within diverse lung tissues? Which fungi are simply in transit, which are coevolved or mutualistic symbionts, and which cause disease? What proportion of debilitating fungal lung infections arise from fungi that were already present in previously healthy individuals? What specific adaptations allow lung-inhabiting fungi to survive in a hostile immune environment? What role do wild vertebrates play in dispersal or as zoonotic reservoirs of these fungi? And finally, do lung fungal communities provide benefits to animal hosts?
